# Physical Activity Reduces the Prevalence of Periodontal Disease: Systematic Review and Meta-Analysis

**DOI:** 10.3389/fphys.2019.00234

**Published:** 2019-03-21

**Authors:** Railson de Oliveira Ferreira, Marcio Gonçalves Corrêa, Marcela Baraúna Magno, Anna Paula Costa Ponte Sousa Carvalho Almeida, Nathália Carolina Fernandes Fagundes, Cassiano Kuchenbecker Rosing, Lucianne Cople Maia, Rafael Rodrigues Lima

**Affiliations:** ^1^Laboratory of Functional and Structural Biology, Institute of Biological Sciences, Federal University of Pará, Belém-Pará, Brazil; ^2^Department of Pediatric Dentistry and Orthodontics, School of Dentistry, Federal University of Rio de Janeiro, Rio de Janeiro, Brazil; ^3^Faculty of Medicine and Dentistry, School of Dentistry, University of Alberta, Edmonton, AB, Canada; ^4^Department of Periodontology, Federal University of Rio Grande do Sul, Porto Alegre, Brazil

**Keywords:** physical activity, PRISMA, periodontal disease, periodontitis, systematic review

## Abstract

**Background:** Regular physical activity boosts several physical capacities and reduces many inflammatory markers of several diseases. In this sense, periodontal disease is a multifactorial inflammatory disease of tooth supporting tissues that has been claimed to trigger processes of systemic alterations. The aim of this systematic review and meta-analysis was to assess the effects of physical activity on periodontal disease.

**Methods:** Observational studies published until August 2018 were searched in online databases (PubMed, Scopus, Web of Science, The Cochrane Library, LILACS, OpenGrey, and Google Scholar) after developing a PECO statement that focused on the comparison between adults that followed a routine of exercises or presented a sedentary lifestyle and its effects on periodontal disease. Searching and data extraction were conducted by following PRISMA guidelines. Registration protocol: CRD42016049661. Quality assessment and risk of bias were analyzed by following Fowkes and Fulton protocol.

**Results:** A total of 512 references were retrieved, while only seven were considered eligible. Two meta-analysis involving the prevalence of periodontal disease and unadjusted/adjusted Odds ratio were performed. One of studies did not find association between clinical periodontal parameters and physical activity. Six articles suggested an association between periodontal disease and regular practice of physical activity since a reduction of periodontal prevalence was observed. Moderate level of evidence was demonstrated on GRADE analysis.

**Conclusion:** Physical activity was associated as a potential tool for reduction of periodontal disease prevalence. The frequency of physical activity is directly related to a low occurrence of periodontitis. However, it is important that further investigations evaluate the effects of other exercise variables, such as volume and intensity, on the periodontal disease prevalence.

## Introduction

Physical activity and exercise are major topics included in human motricity. Exercise is a planned and structured activity which the main purpose is to improve the body capacities, according to the methodological variables of physical training, such as: volume, intensity, frequency, and type of exercise. The physical activity is defined as any bodily movement produced by skeletal muscles that demands an increase of energy expenditure compared to basal levels. Daily living activities, leisure, occupation, sports, active transportation are included as examples of physical activity (Chodzko-Zajko et al., 2009; Garber et al., 2011).

Regular physical activity boosts several physical capacities in general aspects of human health (Garber et al., 2011; Warburton and Bredin, 2017). Physical activity has been related to cognitive improvement (Fernandes et al., 2018b), increased proprioception (Salles et al., 2018) and total lung capacity (McNamara et al., 2018), maintenance of cardiovascular physiology (de Souza et al., 2018), fat reduction (de Souza et al., 2018), and other systemic diseases. The interaction between physical activity and systemic diseases, mainly inflammatory disorders, is still not clearly understood; however, the modulation of immune markers figures as the main potential mechanism involved (Gleeson et al., 2011). Physical activity has been associated with changes in inflammatory markers, including a reduction of Protein C-Reactive levels (Malali et al., 2010; Fernandes et al., 2018a). The increased expression of this protein is related several diseases, including periodontal disease (Beck et al., 2018).

Periodontal disease is the second most prevalent oral disease in humans, since 70% of the global population present one or more damages in periodontal supporting tissues which include: gingiva, periodontal ligament and alveolar bone (Oppermann et al., 2015). Periodontal disease has multifactorial etiology with a marked inflammatory profile and is product of the interaction among bacterial pathogens, host response and individual health habits (American Academy of Periodontology, 2015; Papapanou et al., 2018). In case of gingivitis, signs and symptoms, such as bleeding during flossing, halitosis and swelling, are restricted to marginal gingiva. When the inflammatory signs comprise deeper tissues, such as: gingival bleeding, gingival recession, tooth mobility, destruction of periodontal ligament, alveolar bone resorption, and ultimately tooth loss; periodontitis is present (Papapanou et al., 2018).

Oral hygiene and self-care measures are essential to maintain a good periodontal health. However, other factors, such as the host individual factors, oral environment, and chronic injuries acting as modifying factors of periodontal disease should be considered, since dental plaque represents only 20% of direct risk for periodontal disease development (Lang and Bartold, 2018). In addition, the worsening of periodontal disease may also result in a chronic and systemic inflammation, which is a concept already suggested by other studies that evaluated the inflammatory aspects of different diseases (Potempa et al., 2017; Teixeira et al., 2017), in a possible reciprocal mechanism. Thus, additional procedures to improve professional and personal care may lead to possible adjuvant therapies to periodontal disease.

Physical activity modulates cardiovascular function and is related to prevention, prevalence reduction, and treatment of pathologies involved to this system (Chodzko-Zajko et al., 2009; Garber et al., 2011). Periodontal disease as an inflammatory oral disease may be positively influenced by physical activity. However, it is not clear the relationship between physical activity and periodontal disease. Therefore, a systematic review with meta-analysis was performed in order to answer such question.

## Materials and Methods

### Registration Protocol and Study Design

This systematic review was registered at PROSPERO under the registration code CRD42016049661 and performed according to PRISMA (Preferred Reporting Items for Systematic Review and Meta-Analysis) guidelines (Moher et al., 2009) (Supplementary File 1). Furthermore, this review was conducted following systematic review and meta-analysis elaboration criteria previously proposed by Harris et al. (2014).

### Eligibility Criteria, Search Strategy, and Data Extraction

Observational studies published until September 2018 were searched in online databases after developing a PECO statement that focused on the comparison between adults (Participants) that followed a routine of physical activity (Exposition) or presented a sedentary lifestyle (Comparison) and its effects on Periodontal disease (Outcome). No restriction were applied regarding type of exercise. Only cross-sectional studies, case-control, and cohorts were considered eligible.

PubMed, Scopus, Web of Science, The Cochrane Library, LILACS databases were selected for searches. OpenGrey and Google Scholar were also selected to perform searches on gray literature. Combination of controlled pre-defined MeSh (Medical Subject Headings) and free terms related to physical activity and periodontal disease were used and combined searches with “humans” or “adults” or “exercise” or “physical fitness” and “periodontitis” or “periodontal disease” or “gingivitis.” The search strategy to selected databases is depicted in Supplementary File 2. Differences of database syntax rules were considered to execute combination of pre-defined terms.

A bibliographic reference manager (EndNote, x7 version, Thomson Reuters) was used to save retrieved citations in all databases. Duplicated results were removed and only one publication considered. Articles with titles and abstracts that did not respect our eligibility criteria were also excluded as well as opinion articles, technical articles, guidelines and animal studies. Remaining articles were evaluated and judged by reading their full-texts.

During the full-text analysis, citations were sought from reference lists of all previously selected remaining articles. Two examiners (ROF and MGC) conducted all searches in databases. In case of disagreement, a third examiner (RRL) checked the searches.

### Quality Assessment Analysis

Methodological quality and the risk of bias followed the checklist of Fowkes and Fulton (1991). The analysis comprised study designs and study samples; control group characteristics; quality of measurements and results, completeness; and distortions influences were analyzed among eligible articles respecting domains of the selected protocols (Fowkes and Fulton, 1991; Moher et al., 2009).

Major problems related to analyzed criteria received the sign (++). The sign (+) was applied when the research had a criterion with minor problem. In case of no problems related to criteria, the number “zero” (0) was applied. Not applicable analysis due to the design of study was also checked, but with the sign “NA.” All evaluation criteria are depicted on Supplementary File 3.

### Data Extraction

Selected articles were submitted to extraction of data. Report of the year of publication, study design, participant characteristics (source and sample size), average age, periodontitis evaluation, physical activity evaluation, statistical analysis, and results are presented on Table 1.

**Table 1:** Data extraction of the selected articles.

**References**	**Study design**	**Participants**	**Age**	**Periodontitis diagnosis**		**Exercise evaluation**	**Statistical analysis**	**Results**	**Outcomes**
				**Clinical**	**Laboratorial**	**Clinical**			
Sakki et al., 1995	Cross-sectional	*n* = 527 Male = 266 Female = 261	55 years	Probing depth Periodontal pockets > 3mm	None	Questionaire–Low exercise–Less than 15 min walking, cycling, walking to work and exercised only once or less in a week High – Other practices over low exercise rate	Logistic regression analysis	Lifestyle had an independent association with periodontal health. Periodontal pocketing increased with annanheallhier lifestyle	Lifestyle could explain some of the social and sex differences in periodontal health
Al-Zahrani et al., 2005a	Cross-sectional	*n* = 2,521 Male = 1,245 Female = 1,276	Mean age (SE) Without periodontitis 47.4(0.5) With periodontitis 50.3(0.9)	Probing depth Clinical Attachment loss	None	Questionnaire -nine-leisure time activities - –walking a mile, running, cycling, aerobics exercise, dancing, swimming, calisthenics, garden or yard work and weight lifting	Multivariable logistic regression analysis	Engaging in the recommend level of physical activity (OR 0.58, 95% CI, 0.35–0.96; *p* < 0.05). Smoking, however, was found to modify this relationship. among never (OR 0.46, 95% CI, 0.23–0.93) and former smokers (ORZ 0.26, 95% CI: 0.09–0.72)	Engaging in the recommended level of exercise is associated with lower periodontitis prevalence, especially among never and former smokers
Al-Zahrani et al., 2005b	Cross-sectional	*N* = 12.110	N° enhancing-behaviors Mean age (SE) 0–43.4 (0.45) 1–40.2 (0.64) 2 −39.7 (0.80) 3–48.8 (1.43)	Probing depth Gingival bleeding	None	Comparison Periodontitis Three-enhancing behaviors 1—Questionnaire of nine-leisure time walking mile, running, cycling, aerobics exercise, dancing, swimming, calisthenics, garden or yard work and weight lifting. 2—Maintaining body mass index 3—Dietary quality—Healthy Eating Index (HEI)	Multivariable logistic regression analysis	Engaging in one health behavior is associated with 16% reduction in the prevalence of periodontitis ([OR] = 0.84; 95% [CI] = 0.77-0.93). Engaging Three healthy behaviors were associated with 40% reduction of periodontitis prevalence compared to individuals with none of these health-enhancing behaviors	An increase number of healthy-behaviors is associated with lower periodontitis prevalence
Sanders et al., 2009	Case-control	*n* = 751 Case = 359 Controls = 392	(143)18–44 years (201) 45–64 years (108) 65+ years	Probing depth and Gengival Recession to obtain the Clinical Attachment Loss	GCF e IL-1b (ELISA methods)	Questionnaire-Leisure-time physical activity. Physical activity A sufficiently active group (>150 min over 5 sessions) vs. an insufficiently active group (< 150 min. over 5 sessions).	Unconditional logistic regression	Subjects, with a prescribed threshold for leisure-time physical activity had lower adjusted odds of elevated IL-1b: OR 0.69, (95% CI 0.50–0.94) and detectable CRP: OR 0.70 (0.50–0.98) than less active adults. Physical activity was not associated with periodontitis: OR 1.14 (0.80–1.62)	Leisure-time physical activity may protect against an excessive inflammatory response in periodontitis
Bawadi et al., 2011	Cross-sectional	*n* = 340 Male = 168 Female = 172 Low physical activity (171) Moderate physical activity (108) High physical activity (61)	18–70 years Mean 36± 14.9 years	Plaque index, Gingival Index Probing depth Clinical Attachment Loss	None	Questionnaire- International Physical Activity Questionnaire (IPAQ)	General Linear Model Multivariate procedure. This procedure provided regression analysis and analysis of variance for multiple dependent variables (periodontal parameters) by different explanatory variables and covariates.	Individuals who were highly physically active had a significantly lower PI, GI, CAL and percentage of sites with CAL± 3 mm compared to individuals with a low and moderate level of physical activity	A low physical activity level and a poor diet were significantly associated with increased odds of periodontal disease. Further studies are needed to understand this relationship in greater detail
Merchant et al., 2003	Cause Cohort	*n* = 2123 Men	Age: 35–49 50–54 55–59 60–64 65–69 70+	Radiographic analysis Sub-sample (*n* = 137) Blinded dentist Bone loss (mm) at proximal sites (mesial/distal) with periodontal probe, viewing box and loupes	None	Questionnaire– Hours per week walking (walking to work), hiking, jogging, running, bicycling, using a stationary bicycle, swimming, playing tennis, squash, or aerobics, and the numbers of flights of stairs that participants climbed.	Cox regression analysis–Relative risk and Hazard Ratios Cox regression analysis–Relative risk and Hazard Ratios		A linear inverse association of sustained physical activity and periodontitis was found after confounders adjustment. A healthy lifestyle that incorporates physical activity, including walking, may be beneficial to periodontal health
Samnieng et al., 2013	Cross-sectional	*n* = 612 Males = 158 Female = 454	Mean = 68.8 ± 5.9 years	Probing depth Clinical attachment loss	None	Questionnaire Subjects were dichotomized into a high health practices group (six to seven practices) and a low health practices (zero to five practices) group based on the number of desirable health practices.	ANCOVA analysis.	ANCOVA analysis demonstrated the following significant association: physical activity with periodontal disease and salivary flow rate	Health practices were also associated with better oral health outcomes such as higher number of teeth present, fewer DT, less periodontal disease, oral malodour, and higher salivary flow rate

If absence of information compromised data extraction or risk of bias evaluation, an attempt to contact the authors by e-mail was made. The contact consisted in sending a weekly email, for up to 5 consecutive weeks.

### Risk of Bias

After analysis of checklist domains, including methods and outcomes, summary questions about all analyzed were used to evaluate the risk of bias of included studies. The three summary questions are: Are the results biased? Confounding factors are present on the results? and Is there any possibility of the results occurring by chance?” Examiners applied the answers “YES” and “NO” for each one of the questions. If the article received NO on the three questions, it was considered methodologically feasible, with low risk of bias.

### Quantitative Synthesis (Meta-Analysis)

The extracted data were analyzed using the RevMan software (Review Manager, version 5.3, The Cochrane Collaboration; Copenhagen, Denmark) to assess the relationship between physical activity and periodontal disease.

In the first meta-analysis, the prevalence of Periodontal disease (events) and the total number of individuals in case (sedentary/inactive) and control (physical activity/active) groups were included to calculate the Odds Ratio (OR) with a 95% confidence interval (CI).

In attempt to minimize the impact of confounding factors, a second meta-analysis was constructed including the OR and its 95% CI comparing these two groups (physical activity/active and sedentary/inactive individuals) from included studies and considering a subgroup analysis for adjusted and unadjusted OR. These ratio measures effect as a log OR and the standard error of the log OR using generic inverse-variance weighting method. Combined results were presented as a pooled odds ratio.

Random-effects models were employed due to the fact that the studies were not functionally equivalent in which the objective was to generalize the results from the meta-analysis (Borenstein et al., 2010) and heterogeneity was tested using the *I*^2^ index. Sensitivity analyses were further conducted to estimate and verify the influence of studies, one by one, or grouped, on the pooled results if the heterogeneity was substantial or considerable (50–100%) (Higgins, 2011). The Funnel plot was generated to demonstrate possible publication bias (Mavridis and Salanti, 2014).

Only studies classified as “NO” for Summary Questions were included in quantitative analysis. If some of the information needed for the meta-analysis was absent from any of the selected studies, the authors were contacted to provide the missing data.

### Assessment of Quality of Evidence

The quality of the evidence (certainty in the estimates of effect) was determined for the outcome using the Grading of Recommendations Assessment, Development and Evaluation (GRADE) approach (Ryan, 2016), whereby observational studies start as moderate evidence, and the quality of, or certainty in, the body of evidence decreases to low or very low quality, if serious or very serious issues, related to risk of bias, inconsistency, indirectness, imprecision, and publication bias, are present. In addition, the quality of the evidence can be upgraded if the magnitude of effect is large or very large, or if the effect of all plausible confounding factors would be to reduce the effect, or suggest a spurious effect. In this way, the quality of the evidence can vary from very low to high.

## Results

### Study Selection and Characteristics

**Figure 1:**
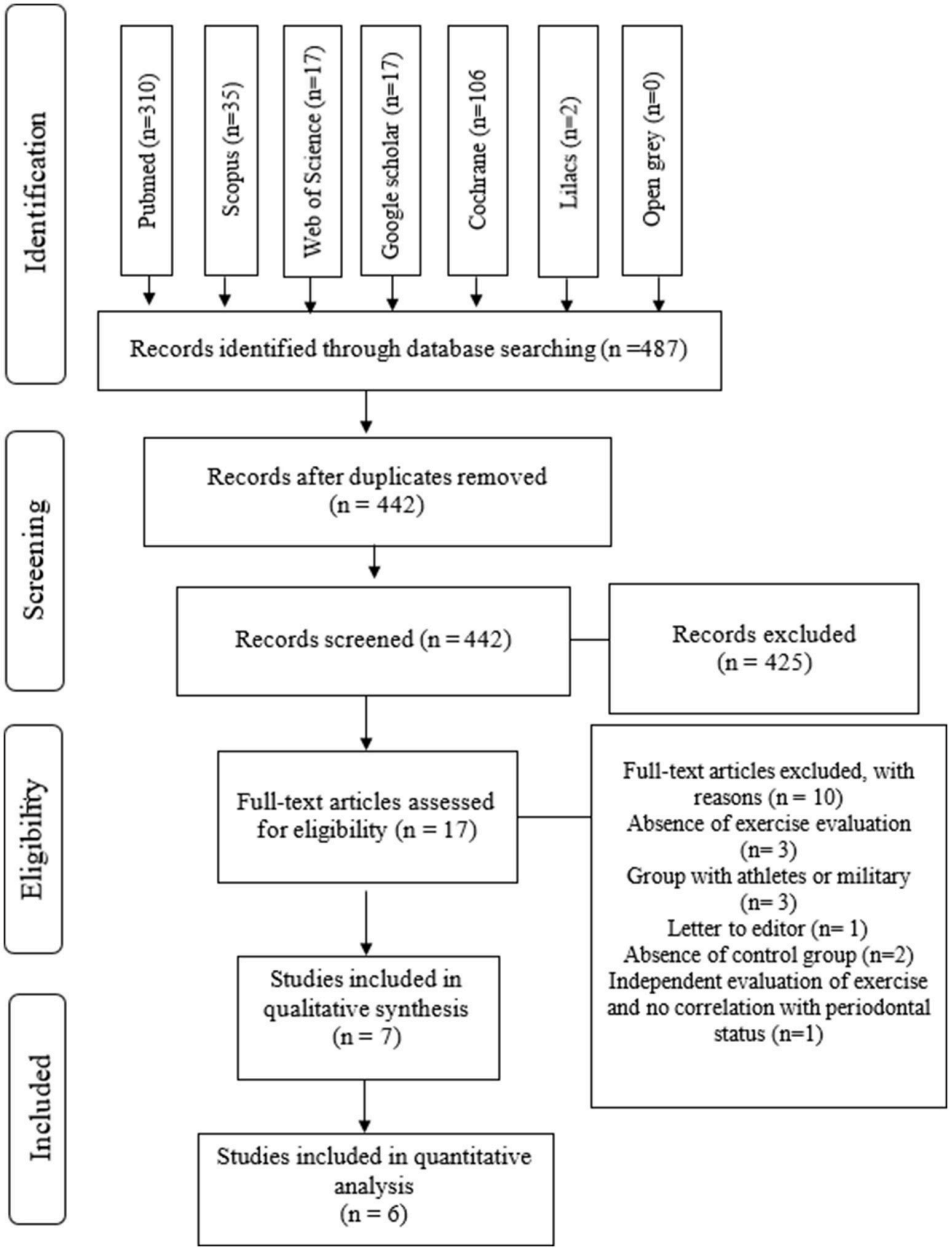
Flow diagram of the screened articles accordingly PRISMA statement.

Among the 512 references identified in searching databases, 45 duplicates were removed. After reading titles and abstracts, 425 out of 442 references were excluded based on the eligibility criteria; thus, 17 references were selected for full text appraisal. Thereafter, three studies were excluded due to the lack of physical activity evaluation (Wakai et al., 1999; Shimazaki et al., 2010; Salekzamani et al., 2011), three studies were excluded since the authors did not evaluate the participants of control groups (Gay-Escoda et al., 2011; Oliveira et al., 2015; D'Ercole et al., 2016), one reference was a letter to editor (Akhter et al., 2008), two studies were not included due to absence of control groups (Hamalainen et al., 2004; Eberhard et al., 2014), and one study was excluded because both evaluations of exercise and periodontitis were independent and not correlated (Ericsson et al., 2016) (See Supplementary File 4). Finally, seven articles were eligible for qualitative assessment (Figure 1).

### Results of Individual Studies

One case-control, one cause cohort, and five cross-sectional studies were included. The association between physical activity and periodontitis were reported in six studies (Sakki et al., 1995; Merchant et al., 2003; Al-Zahrani et al., 2005a,b; Bawadi et al., 2011; Samnieng et al., 2013). The authors attributed the absence of association reported in one study to the characteristics of exercise evaluation (Al-Zahrani et al., 2005b). The average age of participants was around 30–50 years. Dichotomous variables (yes or no/presence or absence) have been used for the evaluation of large samples. Therefore, studies that evaluated periodontitis based on dichotomous variables may be important when considering samples with high number, even if distortion adjustments are needed.

The clinical and oral health/periodontal parameters reported in the selected studies were: number of missing teeth (MT), probing pocket depth (PPD), clinical attachment loss (CAL), loss of attachment (LA), radiographic analysis, recent periodontal surgery, periodontal index (PI), and plaque index (PI). Level of physical activity was measured by means of the questionnaires on the frequency and intensity of physical activity such as the International Physical Activity Questionnaire (IPAQ). Physical activity was self-reported by participants and activities encompassed walking a mile or to work, jogging, running, cycling, aerobics exercise, hiking, dancing, swimming, calisthenics, garden or yard work, and weight lifting.

The practice of physical activity was associated with a low prevalence of periodontitis, which presented an overall 16% reduction in one out of four studies (Al-Zahrani et al., 2005a). In the same study, periodontitis among former smokers was 74% lower for physically active than inactive individuals. Similar results were found in other articles (Sakki et al., 1995; Merchant et al., 2003; Al-Zahrani et al., 2005b; Bawadi et al., 2011), which reported reductions of prevalence around 10% to 16%.

The frequency of physical activity was found to have an influence on periodontitis. Individuals that engaged in the recommended routine of physical activity between 3 and 5 times a week presented lower prevalence of periodontitis in comparison to controls (Sakki et al., 1995; Merchant et al., 2003; Al-Zahrani et al., 2005a,b). One study measured through metabolic equivalent (MET) units per week (one MET is defined as the caloric need per kilogram of body weight for an hour of physical activity divided by the caloric need for the same time spent sitting quietly on a chair) (Merchant et al., 2003) and reported that individuals with higher METs had lower relative risk of periodontitis (~13%).

### Qualitative Synthesis of the Studies

Two out of seven studies utilized sub-samples of United States national surveys (Al-Zahrani et al., 2005a,b) by using a cross-sectional method. For this reason, some studies did not report the data regarding the clinical periodontal parameters (CAL, bleeding on probing, etc.) used for periodontal diagnostic. One study evaluated the participants by using biomarkers in gingival crevicular fluid (Sanders et al., 2009).

Most of problems observed in the studies were related to the lack of adequate sampling methods (Sakki et al., 1995; Merchant et al., 2003; Al-Zahrani et al., 2005a,b), inclusion and exclusion criteria such as participants with smoking habits or systemic diseases (Sakki et al., 1995; Merchant et al., 2003; Al-Zahrani et al., 2005a,b; Samnieng et al., 2013), and small sample size (Sanders et al., 2009).

An association between Periodontal disease and physical activity was found in six out of seven studies (Sakki et al., 1995; Al-Zahrani et al., 2005a,b; Sanders et al., 2009; Bawadi et al., 2011; Samnieng et al., 2013) this link was established by the comparison of frequency and intensity of the exercises (Sakki et al., 1995; Al-Zahrani et al., 2005a,b; Bawadi et al., 2011; Samnieng et al., 2013), reduction on the level of pro-inflammatory factors (Sanders et al., 2009) and regression analysis of population healthcare data (Sakki et al., 1995; Al-Zahrani et al., 2005a,b; Bawadi et al., 2011).

### Risk of Bias

The quality of the measurements reported in each article is shown in Table 2. None of the studies were considered to have an increased risk of bias; however, some issues related to assessment criteria (source of sample, sample size, group matching/randomization, and quality control of the study design) reduced the quality of the generated evidence. All included studies were classified as having a low risk of bias (Sakki et al., 1995; Merchant et al., 2003; Al-Zahrani et al., 2005a,b; Sanders et al., 2009; Bawadi et al., 2011; Samnieng et al., 2013).

**Table 2:** Quality assessment of methods and risk of bias for selected studies.

**Guideline**	**Checklist**	**Sakki et al., 1995**	**Merchant et al., 2003**	**Al-Zahrani et al., 2005b**	**Al-Zahrani et al., 2005a**	**Sanders et al., 2009**	**Bawadi et al., 2011**	**Samnieng et al., 2013**
Study design appropriate to objectives?	Objective common design							
	Prevalence Cross-sectional							
	Prognosis cohort							
	Treatment controlled trial							
	Cause Cohort, case-control, cross-sectional	0	0	0	0	0	0	0
Study sample representative?	Source of sample	0	++	0	0	0	0	0
	Sampling method	++	++	++	+	0	0	0
	Sample size	+	+	+	0	0	+	0
	Entry criteria/exclusion	0	0	0	0	0	0	0
	Non-respondents	0	0	0	0	0	0	0
Control group acceptable?	Definition of controls	0	0	0	0	0	0	0
	Source of controls	0	0	0	0	0	0	0
	Matching/randomization	0	+	+	0	+	0	0
	Comparable characteristics	0	0	0	0	0	0	0
Quality of measurements and outcomes?	Validity	0	0	0	0	0	0	0
	Reproducibility	0	0	0	0	0	0	0
	Blindness	0	0	0	0	0	0	0
	Quality control	+	+	0	0	0	0	0
Completeness	Compliance	0	0	0	0	0	0	0
	Drop outs	0	0	0	0	0	0	0
	Deaths	0	0	0	0	0	0	0
	Missing data	0	0	0	0	0	0	0
Distorting influences?	Extraneous treatments	0	0	0	0	0	0	0
	Contamination	0	0	0	0	0	0	0
	Changes over time	0	0	0	0	0	0	0
	Confounding factors	0	0	0	0	0	0	0
	Distortion reduced by analysis	0	0	0	0	0	0	0
Summary questions	Bias:							
	Are the results erroneously biased in certain direction?	NO	NO	NO	NO	NO	NO	NO
	Confounding:							
	Are there any serious confusing or other distorting influences?	NO	NO	NO	NO	NO	NO	NO
	Chance:							
	Is it likely that the results occurred by chance?	NO	NO	NO	NO	NO	NO	NO

### Quantitative Analysis of the Studies

**Figure 2:**
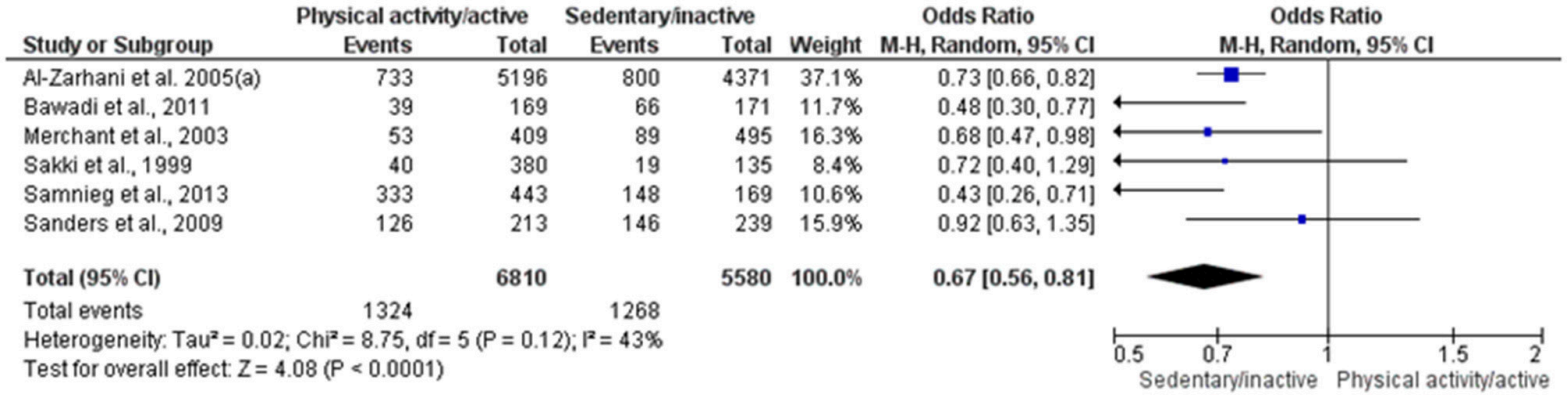
Forest plot of association between physical activity and prevalence of PD.

Six studies with low risk of bias were included in two quantitative syntheses (Sakki et al., 1995; Merchant et al., 2003; Al-Zahrani et al., 2005a; Sanders et al., 2009; Bawadi et al., 2011; Samnieng et al., 2013). One of the seven selected studies was excluded due to a possible overlap of sample (Al-Zahrani et al., 2005b). The first meta-analysis presented moderate heterogeneity (*I*^2^ = 43%) and no statistical significance (*p* = 0.12); thus, no sensitivity analysis was conducted. PD was diagnosed in 19.4% physically active individuals (1,324 out of 6,810) and 22.8% sedentary/inactive individuals (1,268 out of 5,580), which showed a positive association between the analyzed factors (*OR* = 0.67; 95% CI 0.56–0.81; *p* < 0.0001; *I*^2^ = 43%). A sedentary/inactive lifestyle was significantly associated with increased odds of PD (Figure 2). This evidence was qualified as moderate (Table 3) and no publication bias was visually detected related to x- and y- axis's (Supplementary File 5).

**Table 3:** Summary of findings of physical activity/active compared to sedentary/inactive individuals for periodontal disease prevalence.

**Certainty assessment**							**No of patients**		**Effect**		**Certainty**
**No of studies**	**Study design**	**Risk of bias**	**Inconsistency**	**Indirectness**	**Imprecision**	**Other considerations**	**Physical activity/active**	**Sedentary/inactive**	**Relative (95% CI)**	**Absolute (95% CI)**	
**1st META-ANALYSIS. PREVALENCE OF PERIODONTAL DISEASE**											
6	Observational studies	Not serious	Not serious	Not serious	Not serious	All plausible residual confounding would reduce the demonstrated effect	1,324/6,810 (19.4%)	1,268/5,580 (22.7%)	OR 0.67 (0.56 to 0.81)	63 fewer per 1.000 (from 35 fewer to 86 fewer)	⊕⊕⊕○ MODERATE
**2nd META-ANALYSIS. LOG ODDS RATIO**											
6	Observational studies	Not serious	Not serious^a^	Not serious	Not serious	All plausible residual confounding would reduce the demonstrated effect	1,324/6,810 (19.4%)	1,268/5,580 (22.7%)	OR 0.78 (0.65 to 0.93)	41 fewer per 1.000 (from 12 fewer to 67 fewer)	⊕⊕⊕○ MODERATE

*CI, confidence interval; OR, odds ratio*.

a *Although the substantial overall heterogeneity (68%), the subgroup analysis detected that it is related with the presence of adjusted model in statistical model of primary studies. There was not wide variation in the effect estimates across studies. Therefore, the authors did not considered it a serious or very serious inconsistency*.

In the second meta-analysis, overall heterogeneity was substantial (*I*^2^ = 68%) and significant (*p* = 0.008). After sensitivity analysis, heterogeneity ranged from 55 to 74%. The sub-grouped heterogeneity was null or not important and not significant (*I*^2^ unadjusted = 0%; *p* = 0.40/*I*^2^ adjusted = 27%; *p* = 0.25). Therefore, since there was no significant reduction of overall heterogeneity and the sub-grouped heterogeneity was not significant, none of the studies was excluded at this stage.

**Figure 3:**
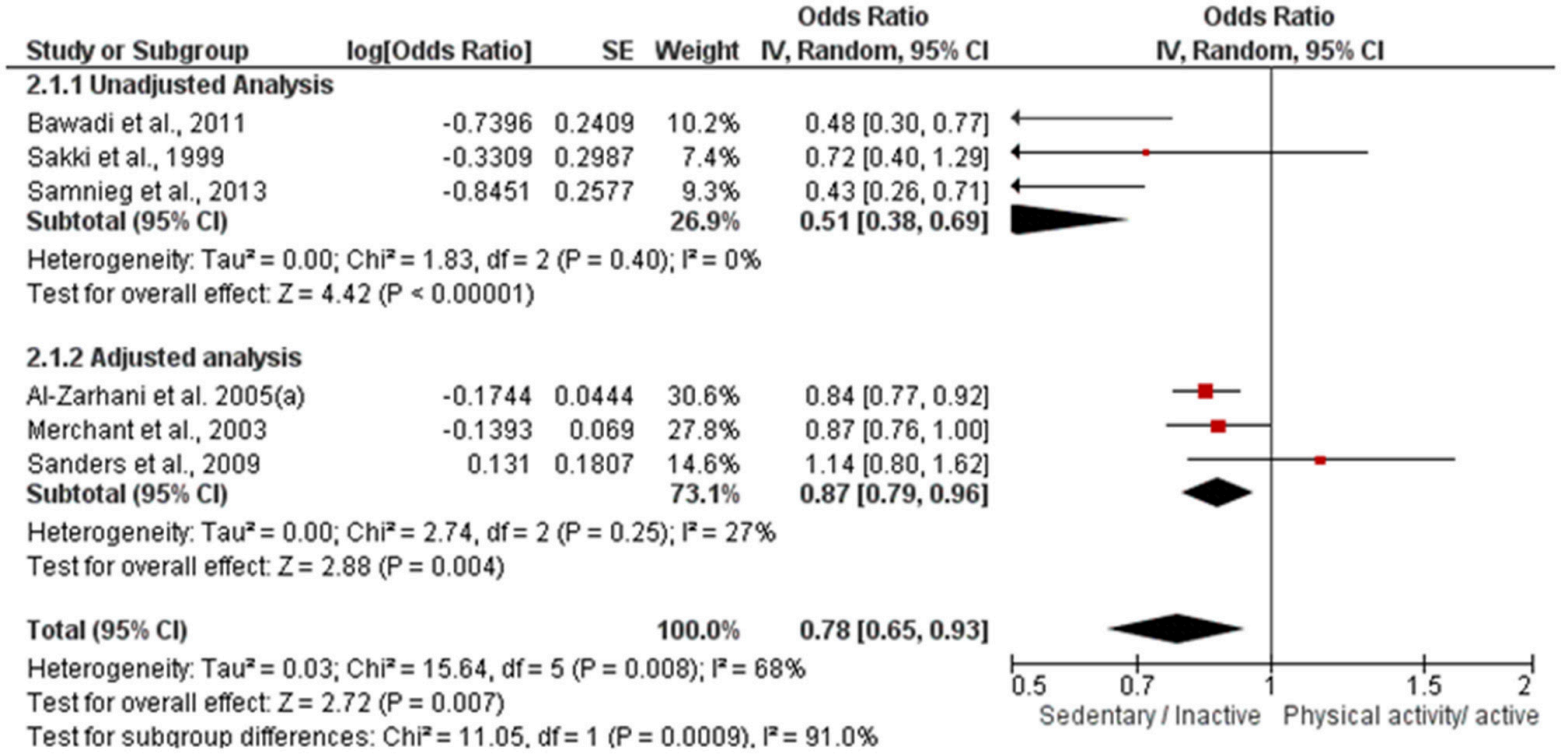
Forest plot of log OR between physical active and periodontal disease.

Data regarding the direction of association between physical activity and PD is show in Figure 3. Physically active individuals were 22% less likely to have PD compared to inactive individuals (*OR* = 0.78; 95% CI 0.65–0.93; *p* = 0.007; *I*^2^ = 68%). The same positive association was observed for subgroup analysis, regardless of model adjustment (OR adjusted = 0.87; 95% CI 0.79–0.96; *p* = 0.004; *I*^2^ = 27%/OR unadjusted = 0.51; 95% CI 0.38–0.69; *p* < 0.00001; *I*^2^ = 0%). This evidence was qualified as moderate. No publication bias was visually detected to x- axis's and some publication bias can be visually detected in y-axis's (See Supplementary File 6). It could be observed that the y-axis's asymmetry is related to statistical adjustment model and standard error, where studies without statistical adjustment present grater standard error and, thus, are located closer to the graphic's base; while studies with statistical adjustment model present lower standard error and are located further away from the graphic's base.

### Summary of Findings and Level of Evidence

Summary of findings analyzed both meta-analysis for prevalence reduction of periodontitis in physical active individuals. Heterogeneity among studies (*I*^2^ = 68%) results on inconsistency problems regarding GRADE analysis. However, absolute and relative effects of inactive and active individuals' analysis, absence of possible publication bias, absence of serious problems among evaluation domains, and reduction of distortions influences results on a moderate level of evidence for outcomes (Table 3).

## Discussion

The findings of the selected studies indicate that physical activity is associated with reduced prevalence of periodontitis. Six articles classified as having a low risk of bias evidenced that physical activity is associated with lower prevalence of periodontitis; this, in comparison to control groups. This association are confirmed by our quantitative analysis and level of evidence assessment.

The synthesis of available data related to scientific questions is essential to select valid information in order to support the decision making of healthcare professionals; thus, systematic reviews fit for that purpose as a tool to summarize the main evidence and to produce missing knowledge (Maia and Antonio, 2012; Harris et al., 2014). Complementing the qualitative synthesis, the meta-analysis performs a synthesis of quantitative data among selected articles estimating the possible effects of the evidence, confirming or denying the hypotheses proposed by the systematic review (Higgins, 2011). The GRADE tool, in turn, analyzes both qualitative and quantitative summaries to promote evidence analysis and subsequent recommendations for evidence-based clinical practice (Ryan, 2016).

Although five articles showed a difference between sedentary and physically active individuals in the prevalence of periodontitis, some issues related to sampling methods and sample size identified during methodological quality assessment should be mentioned. Non-random selection of samples, small sample sizes and mainly group matching/comparisons leads to potential sampling bias and non-representative effect of exercises on periodontal health (Saint-Mont, 2015); therefore, the extrapolation of outcomes statements should be cautious since several factors are involved in the pathology and remission pattern of periodontal diseases.

Otherwise, the restriction of potential confounding factors used in regression analysis is able to estimate which predictor variables support the association between physical activity and periodontal diseases (Petscher and Logan, 2014). Thus, this systematic review presents data that implies the beneficial effects of physical activity on the reduction of periodontal disease prevalence, reduction of inflammatory cytokines and promotion of periodontal health.

Periodontal disease is marked by an inflammatory process as a result of a host response to bacterial infection that leads to destruction of periodontal tissues (gingiva, ligament, alveolar bone) (Papapanou et al., 2018). Health-enhancing habits such as tooth brushing, dental flossing, healthy dietary and regular dental check-ups are important to prevent the progression of Periodontal disease (Al-Zahrani et al., 2005b). The practice of physical activities reported by the selected articles and groups, which figure as another healthy habit, resulted in a 13–16% reduction in the prevalence of Periodontal disease (Al-Zahrani et al., 2005a,b). Theories suggest that this reduction may be related to the effects of exercise on cytokines production and modulation; in this line, studies involving cardiovascular patients have been shown that physical activity modulates several cytokines, in particular the C-reactive protein (CRP) (Sanders et al., 2009; Malali et al., 2010; Fernandes et al., 2018a).

CRP is a by-product of the hepatic metabolism of vitamin K and plays an important role in the physiological cascade of blood coagulation suppression; moreover, increased levels of this inflammatory biomarker are associated with worsening of periodontal condition. Recent studies have shown elevated serum levels of CRP in patients with periodontitis associated or not with other systemic disorders, such as coronary heart disease, stroke, and diabetes mellitus (Beck et al., 2018; Torrungruang et al., 2018). It is very likely that altered levels of systemic biomarkers are associated with worsening of glycemic status; in addition, CRP mediates about 8% of the association between periodontitis and diabetes (Torrungruang et al., 2018).

It is already known that interleukin 1 beta (IL-1β) is directly related to inflammatory levels of several diseases (Sanders et al., 2009; Hashioka et al., 2018). In a recent study, low-to-moderate intensity exercise training during 12 and 24 weeks was able to decrease the levels of IL-1β as well as other pro-inflammatory biomarkers such as IL-6, IL-8, and TNF-α (Hajizadeh Maleki et al., 2018). In this sense, one of the selected studies demonstrated a direct association between physical activity levels and reduction of periodontitis prevalence (Sanders et al., 2009). However, it is important to emphasize that regularity of exercise is a determinant factor for the maintenance of cytokines levels, since it has been demonstrated that these interleukins return to their basal values after 30 days without physical activity (Hajizadeh Maleki et al., 2018).

A low-grade systemic inflammation leads to an imbalance of mechanisms involved in the modulation of serum cytokines levels. IL-10 is a cytokine produced by a variety of cell types (T cells, macrophages, dendritic cells, B cells, monocytes, TCD8+ cells) and presents low levels in individuals with periodontitis (Albuquerque et al., 2012). However, an increase of IL-10 occurs as a response to a transient increase of IL-6, which is resulted from the frequent practice of physical activity. Muscular contraction induces a significant rise of intracellular calcium concentration and consequently increases the production of reactive oxygen species, which in turn increases the synthesis of IL-6. Moreover, IL-10 functions as reducing agent of the expression of several pro-inflammatory cytokines, MHC class II and the co-stimulatory molecules CD80 and CD86 expressed on the surface of antigen-presenting cells.

Intensity, volume and frequency of physical activity are relevant variables observed when investigating the effects of physical activity (Gleeson et al., 2011; Hajizadeh Maleki et al., 2018). Five out of seven selected articles reported an association between the frequency (episodes per week) of physical activity and low prevalence of Periodontal disease. In this line, frequency may be considered the most important variable since five studies reported a 13% reduction of periodontitis prevalence for those individuals with an exercise routine at between 3 and 5 episodes per week. Thus, regularity of physical activity may be necessary to improve periodontal condition and to reduce signs and symptoms through stimulation of pro-inflammatory cytokines (Albuquerque et al., 2012; de Souza et al., 2018; Toker et al., 2018).

In addition to these results, six studies were included in two meta-analyses on periodontitis prevalence (Figures 2, 3). Although the first meta-analysis presented a moderate heterogeneity (*I*^2^ = 43%), the results confirmed that individuals that regularly exercise have lower prevalence of periodontitis than sedentary individuals (*p* < 0.001). The second meta-analysis evaluated the odds ratio between physical activity and the prevalence of periodontitis by means of adjusted and unadjusted results determined by a multivariate analysis. Physically active individuals presented lower prevalence of periodontitis in comparison to control (*p* < 0.0009), albeit a considerable heterogeneity was observed.

Level of evidence evaluation through GRADE guidelines depicted a moderate level to our analysis. GRADE assessment protocol has four levels of recommendations: very low, low, moderate, and high (Ryan, 2016). The moderate result found in this review is directly related to types of studies evaluated, since these present methodologies that are immediately below the methodologies considered gold standard in the pyramid of evidence-based medicine (Maia and Antonio, 2012; Harris et al., 2014). In addition, another important factor to establish the evidence found was the results presented in the two meta-analysis that demonstrated a direct association between physical activity and the reduction of Periodontal disease prevalence. Both meta-analyses presented a moderate level of evidence, which suggests that more studies are needed to establish strong recommendations related to physical activity and periodontal disease (Ryan, 2016).

Some facts should also be highlighted in the results encountered herein. One of them is the fact that both periodontal disease estimates and the independent variables that are related to exercise were assessed in different ways. It should be highlighted that missing teeth was used in the included studies. This is a true endpoint, however, is related to bad overall oral health and not necessarily to periodontal disease. Tooth loss related to dental caries, in populations, is much more a reality (Oppermann et al., 2015). Also, the characteristics of the physical activity variables should be considered as a source of confusion. On the other hand, the included studies are relatively sound and, even though clinically significant differences might not be claimed, the direction of the association is clear: individuals that practice less physical activity tend to demonstrate higher occurrence of periodontal disease.

One intriguing question comes out from the encountered association: what is the biological plausibility of it? One of the possible answers is the fact that physical activity is clearly associated with reduced inflammatory pattern (de Souza et al., 2018). On the other hand, a behavioral approach is also feasible, in which individuals with protective health behavior, such as: maintaining body weight, consuming high quality diet, and physical activity practicing, might have better general health as well as a better periodontal health (Al-Zahrani et al., 2005b). Of course, this has been considered in the analyses of the included studies and is likely that the association is not spurious, but rather real.

### Limitations

The limitations of the present study are related to the database search strategy, which was focused on observational studies; thus, some strategies to reduce bias such as random allocation were not applied. Investigations on the effects of different types of exercises (strength or aerobic) and other variables (volume and intensity) are important to better understand the reduction of Periodontal disease prevalence (Petscher and Logan, 2014; Saltaji et al., 2016). In this line, biases related to randomization and sample allocation have more possibilities to be avoided. However, this type of long-term prospective investigation often involves challenges of sample compliance and high dropout rates along the follow-ups. Moreover, it should be emphasized that almost half of the included studies come from the United States of America. The reason for that is probably because large surveys are performed in the country, focusing both in oral as well as in other systemic problems.

## Conclusion

Although, it was not possible to establish a prevention relationship between physical exercise and periodontal disease, our results showed an association between prevalence reduction of periodontal disease among participants with a practice of physical activity. In addition, the frequency of physical activity is directly related to a low occurrence of periodontitis. Further longitudinal studies are suggested to evaluates a possible cause-effect relationship between both conditions. The effects of physical activity or exercise can also be evaluated simultaneously with a regular periodontal treatment to verify possible changes in clinical periodontal parameters in function of different modalities and variables of exercise. Thus, the clinical relevance concerning this issue might be clearer in order to inform dental care professionals.

## Author Contributions

RF and MC equally contributed to manuscript. RF and MC designed the study and performed the searches, data extraction, quality assessment, analysis of results, and manuscript elaboration. MM, AA, and NF performed analysis of results and manuscript elaboration. MM and LM performed quantitative analysis and manuscript elaboration. CR and RL performed analysis of results and manuscript elaboration.

### Conflict of Interest Statement

The authors declare that the research was conducted in the absence of any commercial or financial relationships that could be construed as a potential conflict of interest.
