# Supplementary material for: Physical Activity Reduces the Prevalence of Periodontal Disease: Systematic Review and Meta-Analysis
Source: Front Physiol. 2019 Mar 21;10:234. doi: 10.3389/fphys.2019.00234 (PMC6438044; doi:10.3389/fphys.2019.00234)
Supplement: Supplementary file 1 [file Data_Sheet_1.docx]

Supplementary Material

**Physical Activity Reduces the Prevalence of Periodontal Disease:**

**Systematic Review And Meta-Analysis**

**Railson de Oliveira Ferreira¹, Marcio Gonçalves Corrêa¹, Marcela Baraúna Magno², Anna Paula Costa Ponte Sousa Carvalho Almeida¹, Nathália Carolina Fernandes Fagundes³, Cassiano Kuchenbecker Rosing^4^, Lucianne Cople Maia², Rafael Rodrigues Lima¹***

**Correspondence:** Corresponding Author: rafalima@ufpa.br

.

# Supplementary Tables

**Supplementary file 1:** PRISMA 2015 (Preference Reporting Requirements for Systematic Review and Meta-Analysis) guidelines checklist

| **Section/topic** | **#** | **Checklist item** | **Information reported** | | **Line number(s)** |
| --- | --- | --- | --- | --- | --- |
|  |  |  | **Yes** | **No** |  |
| **ADMINISTRATIVE INFORMATION** | | | | | |
| **Title** | | | | | |
| Identification | 1a | Identify the report as a protocol of a systematic review |  |  | Page 1. 1-3, Page 2. 7-9 |
| Update | 1b | If the protocol is for an update of a previous systematic review, identify as such |  |  |  |
| **Registration** | 2 | If registered, provide the name of the registry (e.g., PROSPERO) and registration number in the Abstract |  |  | Page 2. 16 Page 3. 24 |
| **Authors** | | | | | |
| Contact | 3a | Provide name, institutional affiliation, and e-mail address of all protocol authors; provide physical mailing address of corresponding author |  |  | Page 1. 4-28 |
| Contributions | 3b | Describe contributions of protocol authors and identify the guarantor of the review |  |  | Page 11. 7-11 |
| **Amendments** | 4 | If the protocol represents an amendment of a previously completed or published protocol, identify as such and list changes; otherwise, state plan for documenting important protocol amendments |  |  | Page 3. 23-27 |
| **Support** | | | | | |
| Sources | 5a | Indicate sources of financial or other support for the review |  |  | Page 11. 15-16 |
| Sponsor | 5b | Provide name for the review funder and/or sponsor |  |  | Page 11. 15-16 |
| Role of sponsor/funder | 5c | Describe roles of funder(s), sponsor(s), and/or institution(s), if any, in developing the protocol |  |  |  |
| **INTRODUCTION** | | | | | |
| **Rationale** | 6 | Describe the rationale for the review in the context of what is already known |  |  | Page 2. 2-9, Page 2. 38-50 Page 3. 1-17 |
| **Objectives** | 7 | Provide an explicit statement of the question(s) the review will address with reference to participants, interventions, comparators, and outcomes (PICO) |  |  | Page 3. 30-39 |
| **METHODS** | | | | | |
| **Eligibility criteria** | 8 | Specify the study characteristics (e.g., PICO, study design, setting, time frame) and report characteristics (e.g., years considered, language, publication status) to be used as criteria for eligibility for the review |  |  | Page 3.30-47 |
| **Information sources** | 9 | Describe all intended information sources (e.g., electronic databases, contact with study authors, trial registers, or other grey literature sources) with planned dates of coverage |  |  | Page 3.34-47 |
| **Search strategy** | 10 | Present draft of search strategy to be used for at least one electronic database, including planned limits, such that it could be repeated |  |  | Page 3.34-39 |
| ***STUDY RECORDS*** | | | | | |
| Data management | 11a | Describe the mechanism(s) that will be used to manage records and data throughout the review |  |  | Page 3. 42-43 |
| Selection process | 11b | State the process that will be used for selecting studies (e.g., two independent reviewers) through each phase of the review (i.e., screening, eligibility, and inclusion in meta-analysis) |  |  | Page 3. 48-50 Page 4. 1-2 |
| Data collection process | 11c | Describe planned method of extracting data from reports (e.g., piloting forms, done independently, in duplicate), any processes for obtaining and confirming data from investigators |  |  | Page 3. 48-49 Page 4. 1-2 |
| **Data items** | 12 | List and define all variables for which data will be sought (e.g., PICO items, funding sources), any pre-planned data assumptions and simplifications |  |  | Page 3. 34-41 |
| **Outcomes and prioritization** | 13 | List and define all outcomes for which data will be sought, including prioritization of main and additional outcomes, with rationale |  |  | Page 3. 34-41 |
| **Risk of bias in individual studies** | 14 | Describe anticipated methods for assessing risk of bias of individual studies, including whether this will be done at the outcome or study level, or both; state how this information will be used in data synthesis |  |  | Page 4. 29-35 |
| ***DATA*** | | | | | |
| **Synthesis** | 15a | Describe criteria under which study data will be quantitatively synthesized |  |  | Page 4. 38-48 Page 5. 1-12 |
|  | 15b | If data are appropriate for quantitative synthesis, describe planned summary measures, methods of handling data, and methods of combining data from studies, including any planned exploration of consistency (e.g., *I* ^2^, Kendall’s tau) |  |  | Page 5.3-9 |
|  | 15c | Describe any proposed additional analyses (e.g., sensitivity or subgroup analyses, meta-regression) |  |  | Page 4. 41-48 Page 5.1-9 |
|  | 15d | If quantitative synthesis is not appropriate, describe the type of summary planned |  |  | Page 5.10-12 |
| **Meta-bias(es)** | 16 | Specify any planned assessment of meta-bias(es) (e.g., publication bias across studies, selective reporting within studies) |  |  |  |
| **Confidence in cumulative evidence** | 17 | Describe how the strength of the body of evidence will be assessed (e.g., GRADE) |  |  | Page 5. 15-24 |

**Supplementary file 2:** Terms used on databases searches.

| Database | Search format |
| --- | --- |
| PUBMED  N=332 | (((((((((((((((((((((((((((((((((((((((((((((((((((((((((((((((((((((((((Periodontitis[MeSH Terms]) OR Periodontitides[Title/Abstract]) OR Pericementitis[Title/Abstract]) OR Pericementitides[Title/Abstract]) OR Periodontal Diseases[MeSH Terms]) OR Disease, Periodontal[Title/Abstract]) OR Diseases, Periodontal[Title/Abstract]) OR Periodontal Disease[Title/Abstract]) OR Parodontosis[Title/Abstract]) OR Parodontoses[Title/Abstract]) OR Pyorrhea Alveolaris[Title/Abstract]) OR Periodontal Atrophy[MeSH Terms]) OR Periodontal Atrophies[Title/Abstract]) OR Atrophy of Periodontium[Title/Abstract]) OR Periodontium Atrophies[Title/Abstract]) OR Periodontium Atrophy[Title/Abstract]) OR Gingivo-Osseous Atrophy[Title/Abstract]) OR Gingivo Osseous Atrophy[Title/Abstract]) OR Gingivo-Osseous Atrophies[Title/Abstract]) OR Alveolar Bone Loss[MeSH Terms]) OR Alveolar Bone Losses[Title/Abstract]) OR Alveolar Process Atrophy[Title/Abstract]) OR Alveolar Process Atrophies[Title/Abstract]) OR Alveolar Resorption[Title/Abstract]) OR Alveolar Resorptions[Title/Abstract]) OR Resorption, Alveolar[Title/Abstract]) OR Resorptions, Alveolar[Title/Abstract]) OR Bone Loss, Periodontal[Title/Abstract]) OR Bone Losses, Periodontal[Title/Abstract]) OR Periodontal Bone Losses[Title/Abstract]) OR Periodontal Bone Loss[Title/Abstract]) OR Periodontal Resorption[Title/Abstract]) OR Periodontal Resorptions[Title/Abstract]) OR Periodontal Resorptions[Title/Abstract]) OR Resorption, Periodontal[Title/Abstract]) OR Alveolar Bone Atrophy[Title/Abstract]) OR Alveolar Bone Atrophy[Title/Abstract]) OR Alveolar Bone Atrophies[Title/Abstract]) OR Bone Atrophies, Alveolar[Title/Abstract]) OR Bone Atrophy, Alveolar[Title/Abstract]) OR Bone Loss, Alveolar[Title/Abstract]) OR Chronic Periodontitis[MeSH Terms]) OR Chronic Periodontitides[Title/Abstract]) OR Periodontitides, Chronic[Title/Abstract]) OR Periodontitis, Chronic[Title/Abstract]) OR Adult Periodontitis[Title/Abstract]) OR Adult Periodontitides[Title/Abstract]) OR Periodontitides, Adult[Title/Abstract]) OR Periodontitis, Adult[Title/Abstract])) OR Gingiva[MeSH Terms]) OR Gums[Title/Abstract]) OR Gum[Title/Abstract]) OR Interdental Papilla[Title/Abstract]) OR Papilla, Interdental[Title/Abstract]) OR Periodontal Index[MeSH Terms]) OR Index, Periodontal[Title/Abstract]) OR Indices, Periodontal[Title/Abstract]) OR Periodontal Indices[Title/Abstract]) OR Periodontal Indexes[Title/Abstract]) OR Indexes, Periodontal[Title/Abstract]) OR Community Periodontal Index of Treatment Needs[Title/Abstract]) OR CPITN[Title/Abstract]) OR Bleeding on Probing, Gingival[Title/Abstract]) OR Gingival Bleeding on Probing[Title/Abstract]) OR Gingival Index[Title/Abstract]) OR Gingival Indices[Title/Abstract]) OR Index, Gingival[Title/Abstract]) OR Indices, Gingival[Title/Abstract]) OR Gingival Indexes[Title/Abstract]) OR Indexes, Gingival[Title/Abstract])) AND ((((((Humans[MeSH Terms]) OR Man, Modern[Title/Abstract]) OR Modern Man[Title/Abstract]) OR Man Taxonomy[Title/Abstract]) OR Homo sapiens[Title/Abstract]) OR Human[Title/Abstract])) AND ((((((((((((((((((((((((((((Exercise[MeSH Terms]) OR Exercises[Title/Abstract]) OR Physical Activity[Title/Abstract]) OR Activities, Physical[Title/Abstract]) OR Activity, Physical[Title/Abstract]) OR Physical Activities[Title/Abstract]) OR Exercise, Physical[Title/Abstract]) OR Exercises, Physical[Title/Abstract]) OR Physical Exercise[Title/Abstract]) OR Physical Exercises[Title/Abstract]) OR Acute Exercise[Title/Abstract]) OR Acute Exercises[Title/Abstract]) OR Exercise, Acute[Title/Abstract]) OR Exercises, Acute[Title/Abstract]) OR Exercise, Isometric[Title/Abstract]) OR Exercises, Isometric[Title/Abstract]) OR Isometric Exercises[Title/Abstract]) OR Isometric Exercise[Title/Abstract]) OR Exercise, Aerobic[Title/Abstract]) OR Aerobic Exercise[Title/Abstract]) OR Aerobic Exercises[Title/Abstract]) OR Exercises, Aerobic[Title/Abstract]) OR Exercise Training[Title/Abstract]) OR Exercise Trainings[Title/Abstract]) OR Training, Exercise[Title/Abstract]) OR Trainings, Exercise[Title/Abstract]) OR Physical Fitness[MeSH Terms]) OR Fitness, Physical[Title/Abstract]) Sort by: Relevance |
| SCOPUS  N=35 | ( ( TITLE-ABS-KEY ( humans ) OR TITLE-ABS-KEY ( man, AND modern ) OR TITLE-ABS-KEY ( modern AND man ) OR TITLE-ABS-KEY ( man AND taxonomy ) OR TITLE-ABS-KEY ( homo AND sapiens ) OR TITLE-ABS-KEY ( human ) ) ) AND ( ( TITLE-ABS-KEY ( exercise ) OR TITLE-ABS-KEY ( exercises ) OR TITLE-ABS-KEY ( "Physical Activity" ) OR TITLE-ABS-KEY ( "Activities, Physical" ) OR TITLE-ABS-KEY ( "Activity, Physical" ) OR TITLE-ABS-KEY ( "Physical Activities" ) OR TITLE-ABS-KEY ( "Exercise, Physical" ) OR TITLE-ABS-KEY ( "Exercises, Physical" ) OR TITLE-ABS-KEY ( "Physical Exercise" ) OR TITLE-ABS-KEY ( "Physical Exercises" ) OR TITLE-ABS-KEY ( "Acute Exercise" ) OR TITLE-ABS-KEY ( "Acute Exercises" ) OR TITLE-ABS-KEY ( "Exercise, Acute" ) OR TITLE-ABS-KEY ( "Exercises, Acute" ) OR TITLE-ABS-KEY ( "Exercise, Isometric" ) OR TITLE-ABS-KEY ( "Exercises, Isometric" ) OR TITLE-ABS-KEY ( "Isometric Exercises" ) OR TITLE-ABS-KEY ( "Isometric Exercise" ) OR TITLE-ABS-KEY ( "Exercise, Aerobic" ) OR TITLE-ABS-KEY ( "Aerobic Exercise" ) OR TITLE-ABS-KEY ( "Aerobic Exercises" ) OR TITLE-ABS-KEY ( "Exercises, Aerobic" ) OR TITLE-ABS-KEY ( "Exercise Training" ) OR TITLE-ABS-KEY ( "Exercise Trainings" ) OR TITLE-ABS-KEY ( "Training, Exercise" ) OR TITLE-ABS-KEY ( "Trainings, Exercise" ) OR TITLE-ABS-KEY ( "Physical Fitness" ) OR TITLE-ABS-KEY ( "Fitness, Physical" ) ) ) AND ( ( TITLE-ABS-KEY ( periodontitis ) OR TITLE-ABS-KEY ( periodontitides ) OR TITLE-ABS-KEY ( pericementitis ) OR TITLE-ABS-KEY ( pericementitis ) OR TITLE-ABS-KEY ( "Periodontal Diseases" ) OR TITLE-ABS-KEY ( "Disease,Periodontal" ) OR TITLE-ABS-KEY ( "Diseases,Periodontal" ) OR TITLE-ABS-KEY ( "Periodontal Disease" ) OR TITLE-ABS-KEY ( parodontosis ) OR TITLE-ABS-KEY ( parodontoses ) OR TITLE-ABS-KEY ( "Pyorrhea Alveolaris" ) OR TITLE-ABS-KEY ( "Periodontal Atrophy" ) OR TITLE-ABS-KEY ( "Periodontal Atrophies" ) OR TITLE-ABS-KEY ( "Atrophy of Periodontium" ) OR TITLE-ABS-KEY ( "Periodontium Atrophies" ) OR TITLE-ABS-KEY ( "Periodontium Atrophy" ) OR TITLE-ABS-KEY ( "Gingivo-Osseous Atrophy" ) OR TITLE-ABS-KEY ( "Gingivo Osseous Atrophy" ) OR TITLE-ABS-KEY ( "Gingivo-Osseous Atrophies" ) OR TITLE-ABS-KEY ( "Alveolar Bone Loss" ) OR TITLE-ABS-KEY ( "Alveolar Bone Losses" ) OR TITLE-ABS-KEY ( "Alveolar Process Atrophy" ) OR TITLE-ABS-KEY ( "Alveolar Process Atrophies" ) OR TITLE-ABS-KEY ( "Alveolar Resorption" ) OR TITLE-ABS-KEY ( "Alveolar Resorptions" ) OR TITLE-ABS-KEY ( "Resorption,Alveolar" ) OR TITLE-ABS-KEY ( "Resorptions,Alveolar" ) OR TITLE-ABS-KEY ( "Bone Loss,Periodontal" ) OR TITLE-ABS-KEY ( "Bone Losses,Periodontal" ) OR TITLE-ABS-KEY ( "Periodontal Bone Losses" ) OR TITLE-ABS-KEY ( "Periodontal Bone Loss" ) OR TITLE-ABS-KEY ( "Periodontal Resorption" ) OR TITLE-ABS-KEY ( "Periodontal Resorptions" ) OR TITLE-ABS-KEY ( "Resorption,Periodontal" ) OR TITLE-ABS-KEY ( "Alveolar Bone Atrophy" ) OR TITLE-ABS-KEY ( "Alveolar Bone Atrophies" ) OR TITLE-ABS-KEY ( "Bone Atrophies,Alveolar" ) OR TITLE-ABS-KEY ( "Bone Atrophy,Alveolar" ) OR TITLE-ABS-KEY ( "Bone Loss,Alveolar" ) OR TITLE-ABS-KEY ( "Chronic Periodontitis" ) OR TITLE-ABS-KEY ( "Chronic Periodontitides" ) OR TITLE-ABS-KEY ( "Periodontitides,Chronic" ) OR TITLE-ABS-KEY ( "Periodontitis,Chronic" ) OR TITLE-ABS-KEY ( "Adult Periodontitis" ) OR TITLE-ABS-KEY ( "Adult Periodontitides" ) OR TITLE-ABS-KEY ( "Periodontitides,Adult" ) OR TITLE-ABS-KEY ( "Periodontitis,Adult" ) OR TITLE-ABS-KEY ( "Periodontal Index" ) OR TITLE-ABS-KEY ( "Index, Periodontal" ) OR TITLE-ABS-KEY ( "Indices, Periodontal" ) OR TITLE-ABS-KEY ( "Periodontal Indices" ) OR TITLE-ABS-KEY ( "Periodontal Indexes" ) OR TITLE-ABS-KEY ( "Indexes, Periodontal" ) OR TITLE-ABS-KEY ( "Community Periodontal Index of Treatment Needs" ) OR TITLE-ABS-KEY ( cpitn ) OR TITLE-ABS-KEY ( "Bleeding on Probing, Gingival" ) OR TITLE-ABS-KEY ( "Gingival Bleeding on Probing" ) OR TITLE-ABS-KEY ( "Gingival Index" ) OR TITLE-ABS-KEY ( "Gingival Indices" ) OR TITLE-ABS-KEY ( "Index, Gingival" ) OR TITLE-ABS-KEY ( "Indices, Gingival" ) AND TITLE-ABS-KEY ( "Gingival Indexes" ) OR TITLE-ABS-KEY ( "Indexes, Gingival" ) OR TITLE-ABS-KEY ( gingivitis ) OR TITLE-ABS-KEY ( gingivitides ) ) ) |
| COCHRANE  N=107 | Humans OR "Man, Modern" OR "Modern Man" OR "Man Taxonomy" OR "Homo sapiens" OR Human AND Exercise OR Exercises OR "Physical Activity" OR "Activities, Physical" OR "Activity, Physical" OR "Physical Activities" OR "Exercise, Physical" OR "Exercises, Physical" OR "Physical Exercise" OR "Physical Exercises" OR "Acute Exercise" OR "Acute Exercises" OR "Exercise, Acute" OR "Exercises, Acute" OR "Exercise, Isometric" OR "Exercises, Isometric" OR "Isometric Exercises" OR "Isometric Exercise" OR "Exercise, Aerobic" OR "Aerobic Exercise" OR "Aerobic Exercises" OR "Exercises, Aerobic" OR "Exercise Training" OR "Exercise Trainings" OR "Training, Exercise" OR "Trainings, Exercise" OR "Physical Fitness" OR "Fitness, Physical" AND Periodontitis OR Periodontitides OR Pericementitis OR Pericementitides OR “Periodontal Diseases” OR “Disease, Periodontal” OR “Diseases, Periodontal” OR “Periodontal Disease” OR Parodontosis OR Parodontoses OR “Pyorrhea Alveolaris” OR “Periodontal Atrophy” OR “Periodontal Atrophies” OR “Atrophy of Periodontium” OR “Periodontium Atrophies” OR “Periodontium Atrophy” OR “Gingivo-Osseous Atrophy” OR “Gingivo Osseous Atrophy” OR “Gingivo-Osseous Atrophies” OR “Alveolar Bone Loss” OR “Alveolar Bone Losses” OR “Alveolar Process Atrophy” OR “Alveolar Process Atrophies” OR “Alveolar Resorption” OR “Alveolar Resorptions” OR “Resorption, Alveolar” OR “Resorptions, Alveolar” OR “Bone Loss, Periodontal” OR “Bone Losses, Periodontal” OR “Periodontal Bone Losses” OR “Periodontal Bone Loss” OR “Periodontal Resorption” OR “Periodontal Resorptions” OR “Periodontal Resorptions” OR “Resorption, Periodontal” OR “Alveolar Bone Atrophy” OR “Alveolar Bone Atrophy” OR “Alveolar Bone Atrophies” OR “Bone Atrophies, Alveolar” OR “Bone Atrophy, Alveolar” OR “Bone Loss, Alveolar” OR “Chronic Periodontitis” OR “Chronic Periodontitides” OR “Periodontitides, Chronic” OR “Periodontitis, Chronic” OR “Adult Periodontitis” OR “Adult Periodontitides” OR “Periodontitides, Adult” OR “Periodontitis, Adult” OR Gingiva OR Gums OR Gum OR “Interdental Papilla” OR “Papilla, Interdental” OR “Periodontal Index” OR “Index, Periodontal” OR “Indices, Periodontal” OR “Periodontal Indices” OR “Periodontal Indexes” OR “Indexes, Periodontal” OR “Community Periodontal Index of Treatment Needs” OR CPITN OR “Bleeding on Probing, Gingival” OR “Gingival Bleeding on Probing” OR “Gingival Index” OR “Gingival Indices” OR “Index, Gingival” OR “Indices, Gingival” OR “Gingival Indexes” OR “Indexes, Gingival” |
| WEB OF SCIENCE  N=17 | TS=(Humans OR "Man, modern" OR "Modern man" OR "Man taxonomy" OR "Homo sapiens" OR Human) AND TS=(Exercise OR Exercises OR "Physical Activity" OR "Activities, Physical" OR "Activity, Physical" OR "Physical Activities" OR "Exercise, Physical" OR "Exercises, Physical" OR "Physical Exercise" OR "Physical Exercises" OR "Acute Exercise" OR "Acute Exercises" OR "Exercise, Acute" OR "Exercises, Acute" OR "Exercise, Isometric" OR "Exercises, Isometric" OR "Isometric Exercises" OR "Isometric Exercise" OR "Exercise, Aerobic" OR "Aerobic Exercise" OR "Aerobic Exercises" OR "Exercises, Aerobic" OR "Exercise Training" OR "Exercise Trainings" OR "Training, Exercise" OR "Trainings, Exercise" OR "Physical Fitness" OR "Fitness, Physical") AND TS=(Periodontitis OR Periodontitides OR Pericementitis OR Pericementitides OR "Periodontal Diseases" OR "Disease, Periodontal" OR "Diseases, Periodontal" OR "Periodontal Disease" OR Parodontosis OR Parodontoses OR "Pyorrhea Alveolaris" OR "Periodontal Atrophy" OR "Periodontal Atrophies" OR "Atrophy of Periodontium" OR "Periodontium Atrophies" OR "Periodontium Atrophy" OR "Gingivo-Osseous Atrophy" OR "Gingivo Osseous Atrophy" OR "Gingivo-Osseous Atrophies" OR "Alveolar Bone Loss" OR "Alveolar Bone Losses" OR "Alveolar Process Atrophy" OR "Alveolar Process Atrophies" OR "Alveolar Resorption" OR "Alveolar Resorptions" OR "Resorption, Alveolar" OR "Resorptions, Alveolar" OR "Bone Loss, Periodontal" OR "Bone Losses, Periodontal" OR "Periodontal Bone Losses" OR "Periodontal Bone Loss" OR "Periodontal Resorption" OR "Periodontal Resorptions" OR "Resorption, Periodontal" OR "Alveolar Bone Atrophy" OR "Alveolar Bone Atrophies" OR "Bone Atrophies, Alveolar" OR "Bone Atrophy, Alveolar" OR "Bone Loss, Alveolar" OR "Chronic Periodontitis" OR "Chronic Periodontitides" OR "Periodontitides, Chronic" OR "Periodontitis, Chronic" OR "Adult Periodontitis" OR "Adult Periodontitides" OR "Periodontitides, Adult" OR "Periodontitis, Adult" OR "Periodontal Index" OR "Index, Periodontal" OR "Indices, Periodontal" OR "Periodontal Indices" OR "Periodontal Indexes" OR "Indexes, Periodontal" OR "Community Periodontal Index of Treatment Needs" OR "CPITN" OR "Bleeding on Probing, Gingival" OR "Gingival Bleeding on Probing" OR "Gingival Index" OR "Gingival Indices" OR "Index, Gingival" OR "Indices, Gingival" OR "Gingival Indexes" OR "Indexes, Gingival" OR Gingivitis OR Gingivitides) |
| CLINICALTRIALS.GOV  N=1 | “Periodontal disease” AND Exercise |
| LILACS  N=2 | Humans OR "Man, Modern" OR "Modern Man" OR "Man Taxonomy" OR "Homo sapiens" OR Human AND Exercise OR Exercises OR "Physical Activity" OR "Activities, Physical" OR "Activity, Physical" OR "Physical Activities" OR "Exercise, Physical" OR "Exercises, Physical" OR "Physical Exercise" OR "Physical Exercises" OR "Acute Exercise" OR "Acute Exercises" OR "Exercise, Acute" OR "Exercises, Acute" OR "Exercise, Isometric" OR "Exercises, Isometric" OR "Isometric Exercises" OR "Isometric Exercise" OR "Exercise, Aerobic" OR "Aerobic Exercise" OR "Aerobic Exercises" OR "Exercises, Aerobic" OR "Exercise Training" OR "Exercise Trainings" OR "Training, Exercise" OR "Trainings, Exercise" OR "Physical Fitness" OR "Fitness, Physical" AND Periodontitis OR Periodontitides OR Pericementitis OR Pericementitides OR “Periodontal Diseases” OR “Disease, Periodontal” OR “Diseases, Periodontal” OR “Periodontal Disease” OR Parodontosis OR Parodontoses OR “Pyorrhea Alveolaris” OR “Periodontal Atrophy” OR “Periodontal Atrophies” OR “Atrophy of Periodontium” OR “Periodontium Atrophies” OR “Periodontium Atrophy” OR “Gingivo-Osseous Atrophy” OR “Gingivo Osseous Atrophy” OR “Gingivo-Osseous Atrophies” OR “Alveolar Bone Loss” OR “Alveolar Bone Losses” OR “Alveolar Process Atrophy” OR “Alveolar Process Atrophies” OR “Alveolar Resorption” OR “Alveolar Resorptions” OR “Resorption, Alveolar” OR “Resorptions, Alveolar” OR “Bone Loss, Periodontal” OR “Bone Losses, Periodontal” OR “Periodontal Bone Losses” OR “Periodontal Bone Loss” OR “Periodontal Resorption” OR “Periodontal Resorptions” OR “Periodontal Resorptions” OR “Resorption, Periodontal” OR “Alveolar Bone Atrophy” OR “Alveolar Bone Atrophy” OR “Alveolar Bone Atrophies” OR “Bone Atrophies, Alveolar” OR “Bone Atrophy, Alveolar” OR “Bone Loss, Alveolar” OR “Chronic Periodontitis” OR “Chronic Periodontitides” OR “Periodontitides, Chronic” OR “Periodontitis, Chronic” OR “Adult Periodontitis” OR “Adult Periodontitides” OR “Periodontitides, Adult” OR “Periodontitis, Adult” OR Gingiva OR Gums OR Gum OR “Interdental Papilla” OR “Papilla, Interdental” OR “Periodontal Index” OR “Index, Periodontal” OR “Indices, Periodontal” OR “Periodontal Indices” OR “Periodontal Indexes” OR “Indexes, Periodontal” OR “Community Periodontal Index of Treatment Needs” OR CPITN OR “Bleeding on Probing, Gingival” OR “Gingival Bleeding on Probing” OR “Gingival Index” OR “Gingival Indices” OR “Index, Gingival” OR “Indices, Gingival” OR “Gingival Indexes” OR “Indexes, Gingival” |
| GOOGLE SCHOLAR  N=17 | "chronic periodontitis" + "physical exercise" + "gingivitis" "-in vitro" “-systematic review” -book |
| OPEN GREY  N=0 | “Periodontal disease” AND Exercise |

**Supplementary file 3**. Assessment criteria for quality of methods and risk of bias

| **Guideline** | **Checklist** | **Description** |
| --- | --- | --- |
| Study design apropriate to objectives? | objective common design | The type of study was marked in the appropriate type of study. If the type of study was appropriate according to the study design was marked as "0" and as "++" if it was not appropriate. |
|  | prevalence Cross-sectional |  |
|  | Prognosis Cohort |  |
|  | Treatment Controled trial |  |
|  | Cause Cohort, case-control, cross-sectional |  |
| Study sample representative? | Source of sample | The domain was considered (0) in cases of detailed origin, (+) to specified origin of only one group and (++) in cases of absence of specification of the origin of the groups |
|  | Sampling method | The item was assigned (0) for full description of sampling method, (+) for poor or no description of sample method, with no problem in matching between groups and (++) for poor or no description of sample method, interfering in matching of the groups |
|  | Sample size | A minor problem (+) was considered when the sample was not representative or did not report a sample calculation. To a major problem, (++) was considered when no sample calculation was provided and the number of participants was less than 50 participants, (0) was considered in absence of the above factors. |
|  | Entry criteria/exclusion | A minor problem (+) was attributed when the control and case group reported a current use of antibiotics or anti-inflammatories, diabetes, smoking or pregnancy. In the case of presence of more than two previously mentioned items was considered as a major problem (++). |
|  | Non-respondents | The (0) was attributed when there was no refusal to participation in the study, (+) was assigned when there was refusal, but did not compromise the sample, and (++) when there was refusal and impairment of the sample size. |
| Control group acceptable? | Definition of controls | It was attributed (0) when all characteristics of control group were described, (+) when any information was pendent as the origin of control group, the selection criterions and a different origin between case and control groups and (++) when two or more items described in previously items. |
|  | Source of controls | It was considered (0) when control group was referred, (+) when the origin of groups was different, but with reasons and (++) when the groups present different origins without reasons. |
|  | Matching/randomization | In this item, (0) was assigned to cases of randomized/matched groups, (+) to cases of no description of randomization, but with matching of groups and (++) to no description of randomization or matching. |
|  | Comparable characteristics | It was attributed (0) to matched groups or not matched by the impossibility of being subsequently adjusted and (++) presence of unpaired variables that were not paired or adjusted. |
| Quality of measurements and outcomes? | Validity | It was considered (0) when the evaluation method applied is appropriate; (+) when using a single method, but with appropriate sensitivity with good specificity; (++) when using a single method, without an adequate specificity or good sensitivity. |
|  | Reproducibility | It was considered (0) whether the evaluation methods were well described; (+) when a lack description of any step of the method was presented, for example, the identification of the patients of the groups studied in laboratory samples, evaluations at different times or application of different methods between groups of certain pathology; (++) when two or more of the previous items are present. |
|  | Blindness | The condition of the study participants was considered to be "Blind", in this case being assigned the signal (0), in cases of "not blind" the signal (++) was attributed. |
|  | Quality control | it was considered a problem when the evaluators were not calibrated; when ungraduated students carried out the assessment without supervision of a qualified dentist; analysis of periodontitis only radiographic and/or depth of bag, to evaluate less than three dental faces or not to mention how many faces were evaluated. When two of these problems were identified, it was considered as a minor problem (+) and major problems (++) if more than two of these characteristics were described. |
| Completeness | Compliance | It was assigned (0) for a sample size that remains the same from the beginning to the end or decreases without compromising the power of the test; (+) for differences in sample size at the end of the study, compromising the power of the test, but with reasons and adjusts; (++) for difference in sample size at the end of the study, compromising the power of the test, without reasons. |
|  | Drop outs | The (0) was scored when there is no loss during the study, (+) when there is withdrawal that involves the inclusion criteria, such as age, sex, (++) when there is withdrawal and it compromises more than one criterion. |
|  | Deaths | This item was scored as Not Applicable (NA), due the type of PECO strategy. |
|  | Missing data | In this item, (0) was assigned to cases of randomized/matched groups, (+) to cases of no description of randomization, but with matching of groups and (++) to no description of randomization or matching. |
| Distorting influences? | Extraneous treatments | In this item, (0) was considered when there were no external influences; (+) when there are external influences, but that does not interfere in the results; (++) when there are external influences and interferes with the results. |
|  | Contamination | This item was scored as Not Applicable (NA), due the type of PECO strategy. |
|  | Changes over time | In this item, (0) was attributed to data collected in the same time period; (+) to data collected from the control group and the study group at different times that may cause distortions; (++) when the previous item was associated with data from studies already published. |
|  | Confounding factors | A problem was considered in case of the presence of men and women under the age of 45, menopausal, smoker, diabetic and obese women. A "minor" (+) problem was attributed when 1 or 2 of these characteristics were present and a "larger" (++) problem if there were 3 or more. |
|  | Distortion reduced by analysis | It was considered (0) when it cites the adjustments of the covariates that present distortions; (+) when the article report adjustment, but does not say the criteria; (++) when a distortions was identified, without adjustment. |
| Summary questions | Bias: Are the results erroneously biased in certain direction? | YES or "NO" answers were assigned for each question. If the answer is NO at the three questions, the article is considered reliable, with low risk of bias. |
|  | Confounding: Are there any serious confusing or other distoring influences? |  |
|  | Chance: Is it likely that the results ocurred by chance? |  |

**Supplementary file 4:** Articles excluded after reading in full and reasons for exclusions

| Reference | Reason for exclusion |
| --- | --- |
| Wakai K, Kawamura T, Umemura O, Hara Y, Machida J, Anno T, Ichihara Y, Mizuno Y, Tamakoshi A, Lin Y, Nakayama T, Ohno Y. Associations of medical status and physical fitness with periodontal disease. J Clin Periodontol. 1999 Oct;26(10):664-72. | Absence of exercise evaluation |
| Salekzamani Y, Shirmohammadi A, Rahbar M, Shakouri SK, Nayebi F. Association between Human Body Composition and Periodontal Disease. ISRN Dent. 2011;2011:863847. doi: 10.5402/2011/863847. Epub 2011 Nov 2. | Absence of exercise evaluation |
| Shimazaki Y, Egami Y, Matsubara T, Koike G, Akifusa S, Jingu S, Yamashita Y. Relationship between obesity and physical fitness and periodontitis. J Periodontol. 2010 Aug;81(8):1124-31. doi: 10.1902/jop.2010.100017. | Absence of exercise evaluation |
| D'Ercole S, Tieri M, Martinelli D, Tripodi D. The effect of swimming on oral health status: competitive versus non-competitive athletes. J Appl Oral Sci. 2016 Apr;24(2):107-13. doi: 10.1590/1678-775720150324. | Absence of control group |
| Gay-Escoda C, Vieira-Duarte-Pereira DM, Ardèvol J, Pruna R, Fernandez J, Valmaseda-Castellón E. Study of the effect of oral health on physical condition of professional soccer players of the Football Club Barcelona. Med Oral Patol Oral Cir Bucal. 2011 May 1;16(3):e436-9. | Absence of control group |
| Oliveira JA, Hoppe CB, Gomes MS, Grecca FS, Haas AN. Periodontal disease as a risk indicator for poor physical fitness: a cross-sectional observational study. J Periodontol. 2015 Jan;86(1):44-52. doi: 10.1902/jop.2014.140270. | Absence of control group |
| Akhter R, Hassan NM, Moriya S, Kashiwazaki H, Inoue N, Morita M. Relationship between periodontal status and physical fitness in an elderly population of nonsmokers in Bangladesh. J Am Geriatr Soc. 2008 Dec;56(12):2368-70. doi: 10.1111/j.1532-5415.2008.02036.x. | Letter to Editor |
| Hämäläinen P1, Rantanen T, Keskinen M, Meurman JH. Oral health status and change in handgrip strength over a 5-year period in 80-year-old people. Gerodontology. 2004 Sep;21(3):155-60. | Absence of control group |
| Eberhard J, Stiesch M, Kerling A, Bara C, Eulert C, Hilfiker-Kleiner D, Hilfiker A, Budde E, Bauersachs J, Kück M, Haverich A, Melk A, Tegtbur U. Moderate and severe periodontitis are independent risk factors associated with low cardiorespiratory fitness in sedentary non-smoking men aged between 45 and 65 years. J Clin Periodontol. 2014 Jan;41(1):31-7. doi: 10.1111/jcpe.12183. Epub 2013 Nov 13. | Absence of control group |
| Ericsson JS, Wennström JL, Lindgren B, Petzold M, Östberg AL, Abrahamsson KH. Health investment behaviours and oral/gingival health condition, a cross-sectional study among Swedish 19-year olds. Acta Odontol Scand. 2016;74(4):265-71. doi: 10.3109/00016357.2015.1112424. Epub 2015 Nov 24. | Independent evaluation of exercise and no correlation with periodontal status |

## Supplementary Figures


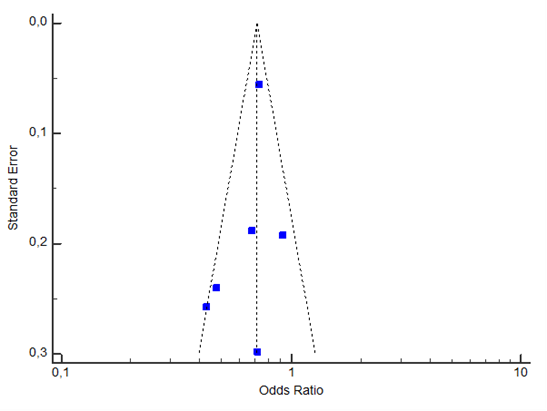


**Supplementary file 5.** Funnel plot of publication bias of studies included in OR meta-analysis.


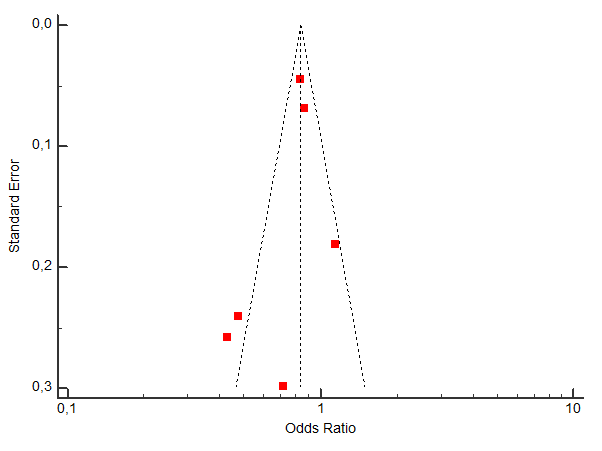


**Supplementary file 6.** Funnel plot of publication bias of studies included in log OR meta-analysis.
